# Epidemiology, clinical characteristics, and risk factors of plastic bronchitis caused by severe Mycoplasma pneumoniae pneumonia in children: a retrospective study from Suzhou, China

**DOI:** 10.3389/fped.2026.1772693

**Published:** 2026-03-09

**Authors:** Lingzhi Ping, Xuena Xu, Xiaowei Zhang, Xiuquan Yang, Yuanyuan Wang, Jia Zhang, Hongjuan Zhang, Chuangli Hao

**Affiliations:** 1 Department of Pediatrics, Xiangcheng District People’s Hospital, Suzhou, China; 2 Department of Respiratory Medicine, Children’s Hospital of Soochow University, Suzhou, China; 3 Department of Pediatrics, The Affiliated Suqian First People’s Hospital of Nanjing Medical University, Suqian, China; 4 Nanjing University of Traditional Chinese Medicine Suqian Affiliated Hospital, Suqian, China

**Keywords:** children, clinical features, plastic bronchitis, risk factors, severe mycoplasma pneumoniae

## Abstract

**Background:**

We analyzed the prevalence and clinical characteristics of children with plastic bronchitis (PB) caused by severe Mycoplasma pneumoniae (SMPP) and explored its risk factors.

**Methods:**

This retrospective study included pediatric patients with SMPP who were admitted to the Respiratory Department of Children’s Hospital of Soochow University and underwent fiberoptic bronchoscopy (FB) treatment between January 1 and December 31, 2024. The SMPP patients were divided into a PB group and a non-PB group according to whether there was a plastic shape under FB. Epidemiological characteristics, general information, clinical manifestations, laboratory findings, imaging features, and treatment regimens were collected and compared between the two groups. Risk factors for PB were identified using logistic regression analysis, and their predictive value was assessed with receiver operating characteristic (ROC) curves.

**Results:**

This study incorporated a total of 510 children diagnosed with SMPP, with 60 and 450 assigned to the PB and non-PB groups, respectively. The epidemic peak of SMPP occurred in summer and autumn; the highest detection rate of PB was recorded in winter (19.30%), with the PB positivity rate peaking in December (32.26%). In the PB group, fever days, runny nose, diminished breath sounds, abnormal liver function, abnormal coagulation function, number of bronchoscopic interventions, neutrophil percentage, C-reactive protein (CRP), alanine aminotransferase (ALT), aspartate aminotransferase (AST), lactate dehydrogenase (LDH), D-dimer, atelectasis, and pleural effusion were all significantly higher compared to the non-PB group (*P* < 0.05). In the PB group, lymphocyte percentage and platelet count were significantly lower compared to the non-PB group (*P* < 0.05). Multivariate logistic regression analysis identified LDH, pleural effusion, and length of hospital stay as independent predictors of PB in children. The combination of these three indicators yielded a notably higher predictive value, with an area under the receiver operating characteristics curve (AUC) of 0.911 (95% CI: 0.868∼0.953).

**Conclusion:**

LDH, pleural effusion, and length of hospital stay were independent risk factors for PB in SMPP children. For children suspected of PB, pediatricians should pay close attention to the above indicators, strive for early diagnosis and treatment, and improve prognosis.

## Background

Mycoplasma pneumoniae (MP) is one of the most common pathogens responsible for community-acquired pneumonia (CAP) in children, particularly among school-aged individuals (1, 2). The detection rate of MP among Chinese children with CAP ranges from 10% to 30% (3). Notably, the detection rate of MP in pediatric respiratory infections has risen to 45.3% in the post-COVID-19 pandemic period (4). Mycoplasma pneumoniae pneumonia (MPP) predominantly affects children over five years of age, with fever and cough as the primary clinical manifestations, which may be accompanied by upper respiratory symptoms such as rhinorrhea, sore throat, or earache (5, 6). Based on clinical manifestations, MPP can be categorized into general MPP and severe MPP (SMPP). SMPP typically manifests around one week into the disease course with both pulmonary and extrapulmonary complications, and carries a high risk of progression to life-threatening illness (7), imposing a substantial dual burden on families and the healthcare system. The incidence of SMPP has shown an increasing trend in recent years, which is closely associated with macrolide antibiotic resistance, dysregulated immune responses, and co-infections (8, 9).

Plastic bronchitis (PB) is a severe complication of SMPP, pathologically defined by the formation of bronchial casts composed primarily of fibrin and mucin, which cause airway obstruction and inflammation (10). This obstruction and inflammation can progress to dyspnea, respiratory failure, and significantly worsen clinical outcomes in children (10, 11). Currently, the pathological classification proposed by Seear et al. remains the standard reference, which categorizes PB into two types: Type I (inflammatory), characterized by abundant inflammatory cells (such as neutrophils and eosinophils) and fibrin; and Type II (non-inflammatory), predominantly composed of mucin and fibrin (12). Respiratory tract infection is an important associated factor for Type I PB, with MP being a particularly common pathogen in this context (13, 14). In clinical practice, fiberoptic bronchoscopy (FB) with bronchoalveolar lavage (BAL) is central to both the diagnosis and management of PB, enabling direct visualization, cast removal, and restoration of airway patency (15).

Currently, there is limited research on risk factors for PB secondary to SMPP in children. Moreover, due to its insidious onset, PB is prone to being overlooked or misdiagnosed. Therefore, systematic identification of risk factors and the establishment of an early warning system are essential for enabling precise interventions and improving clinical outcomes. This study retrospectively analyzes the clinical characteristics of children with SMPP complicated by PB, with the aim of identifying independent risk factors for PB development. The findings are intended to facilitate early recognition of high-risk patients and support timely intervention with an evidence-based approach.

## Methods

### Study population

**Figure 1:**
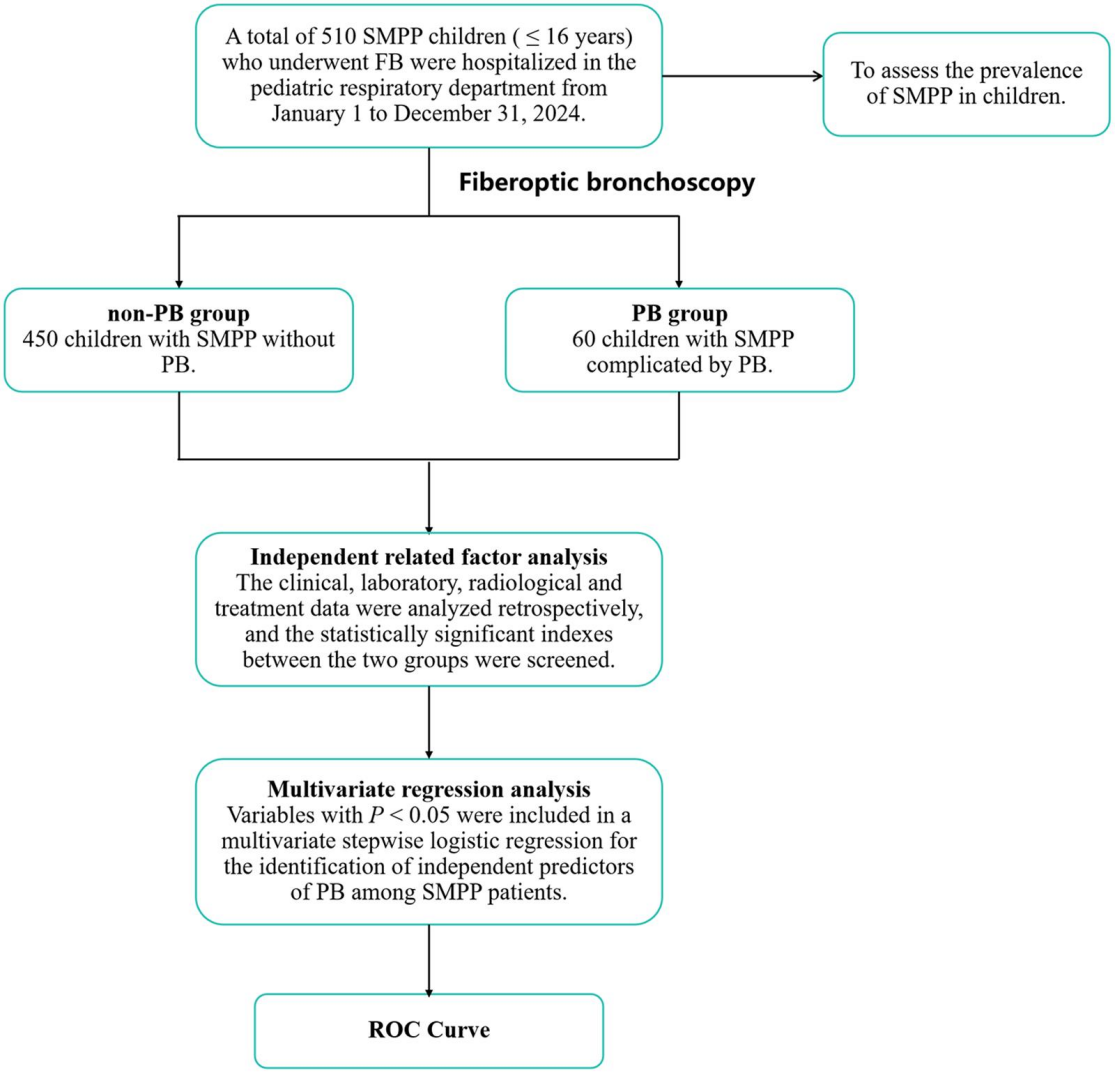
Flowchart of patient selection and statistical analysis.

This retrospective study included pediatric patients with SMPP who were admitted to the Respiratory Department of Children's Hospital of Soochow University between January 1 and December 31, 2024 (Figure 1).

Inclusion criteria were as follows:

(1) Hospitalized children aged between 1 month and 16 years; (2) Diagnosis of CAP established according to the 8th edition of Zhu Futang's Practical Pediatrics, which requires the presence of respiratory symptoms with or without fever, along with chest imaging demonstrating nonspecific patchy pulmonary infiltrates, atelectasis, or consolidation; (3) The positive criteria for MP laboratory diagnosis are to meet any of the following conditions: ① The serum MP antibody titer during the recovery period is increased by four times or more compared to the acute period, ② MP positivity was defined as a positive culture or PCR detection of MP-DNA/RNA in respiratory samples, including throat swab, sputum, or bronchoalveolar lavage fluid(BALF); (4) The diagnostic criteria for SMPP refer to the Diagnosis and Treatment Guidelines for MP in Children (2023 edition) (16); (5) Received FB treatment upon admission; (6) PB was defined by FB findings of airway occlusion with mucus plugs and the retrieval of casts exhibiting a dendritic, bronchial tree-like architecture (12).

Exclusion criteria were as follows:

(1) Underlying comorbidities or immunodeficiencies, including recurrent respiratory tract infections, asthma, chronic lung disease, status post cardiac surgery, severe hematological disorders, congenital or acquired immunodeficiency, and connective tissue diseases; (2) Foreign bodies or non-infectious causes: inhaled foreign bodies, bronchial foreign bodies, aspiration pneumonia; (3) Concurrent infections: co-infection with other pathogens, tuberculosis, or mixed infection with MPP; (4) Disease course or imaging: currently in the SMPP recovery phase or chest x-ray showing no signs of pneumonia; (5) Incomplete data, such as missing hospitalization records.

Patients were classified into PB and non-PB groups based on the identification of branch-like casts during FB. In the PB group, the mucus plugs extracted by lavage, suction, and biopsy forceps could expand into branched casts in saline, while in the non-PB group, no mucus plugs or bronchial obstruction were observed in the trachea, which presented only with mucosal congestion, edema, and mucous sputum.

### Data collection

Patient clinical data were retrospectively extracted from the institutional electronic medical record system. Collected general information comprised hospitalization identification (ID), sex, age, season of onset, and admission date. Medical history covered rhinitis, wheezing, and allergic disorders. Documented clinical manifestations included the duration of fever and cough, presence of dyspnea, pulmonary signs, and extrapulmonary involvement. Laboratory parameters assessed within 24 h of admission consisted of white blood cell (WBC) count, neutrophil percentage, lymphocyte percentage, eosinophil count and percentage, platelet count, C-reactive protein (CRP), alanine aminotransferase (ALT), aspartate aminotransferase (AST), lactate dehydrogenase (LDH), fibrinogen, and D-dimer levels. Relevant pulmonary imaging indicators were also included in the study. Treatment-related variables recorded were the number of bronchoscopy procedures performed and the total hospital stay duration.

### Electronic bronchoscopy

FB with BAL was performed in SMPP pediatric patients with clinical indications, including suspected atelectasis or airway obstruction. Prior to FB, all pediatric patients fasted for 6–8 h. Informed consent was obtained from all guardians by experienced physicians after a comprehensive explanation of the procedure. Subsequently, bronchoscopic examination with BAL was performed, during which BALF samples were collected. All procedures, including bronchoscopy and BALF collection, were conducted in strict compliance with the Chinese Guidelines for Pediatric Flexible Bronchoscopy (17). All BALF samples were subjected to MP-DNA analysis.

### Statistical analysis

The database was established and analysed using SPSS 25.0 (SPSS Inc., Chicago, IL, USA), and GraphPad Prism 9 (GraphPad Software, San Diego, CA). Normally distributed continuous data are expressed as mean ± standard deviation, and comparisons between groups were performed using t-tests. Non-normally distributed continuous data are presented as median (interquartile range), and group comparisons were made using the Mann–Whitney U test. Categorical data are reported as number (percentage), and comparisons between groups were conducted using the Chi-squared test or Fisher's exact test, as appropriate. Variables with *P* < 0.05 in univariate analysis and clinical relevance were included in a multivariate logistic regression model, with the development of plastic bronchitis as the dependent variable. Continuous variables were dichotomized based on their upper and lower quartile values to determine independent predictive factors. Receiver operating characteristic (ROC) curve analysis was employed to evaluate the predictive and diagnostic performance of various indicators for PB in children with SMPP. Statistical significance was set at *P* < 0.05.

## Results

### General information and prevalence of SMPP in children

**Figure 2:**
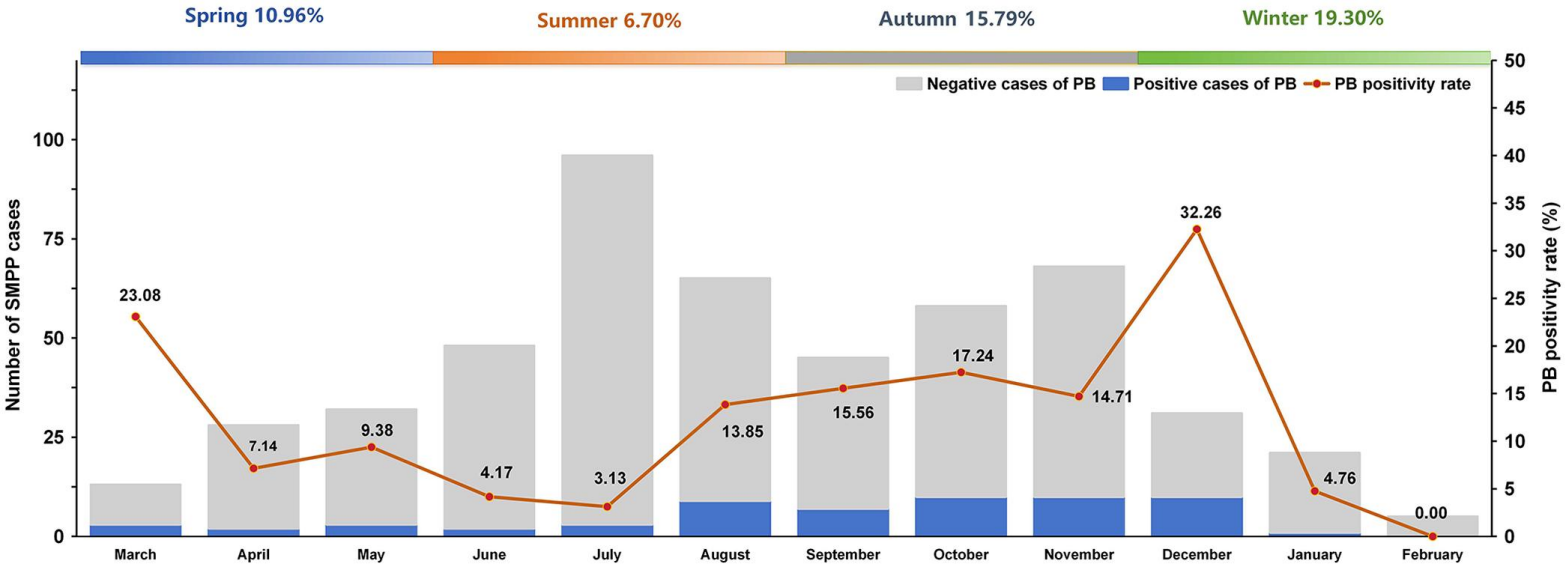
The prevalence of SMPP in children.

This study included data from 510 pediatric patients with SMPP who were admitted to the Respiratory Department of Children's Hospital of Soochow University from January to December 2024. The cohort comprised 450 patients (88.24%) in the non-PB group and 60 patients (11.76%) in the PB group. No significant differences were observed between the two groups in terms of gender or age (*P* > 0.05). In the non-PB and PB groups, male children accounted for 50.22% (226/450) and 40.00% (24/60) of the patients, respectively.Children aged 6–16 years accounted for 73.56% (331/450) of the non-PB group and 80.00% (48/60) of the PB group, representing the highest-risk population for SMPP. Summer accounted for the highest proportion of SMPP hospitalizations (40.98%, 209/510). Seasonal distribution differed between groups, with the non-PB group peaking in summer (43.33%, 195/450) while the PB group peaked in autumn (45.00%, 27/60). A statistically significant difference was found between the groups with respect to the season of onset (*P* < 0.05) (Table 1). Results indicate that the peak incidence of pediatric SMPP primarily occurs during summer and autumn. PB caused by SMPP in children occurred throughout the year, with detection rates of 10.96% in spring, 6.70% in summer, 15.79% in autumn, and 19.30% in winter. The highest number of SMPP cases was observed in July (*n* = 96), with the lowest in February (*n* = 5). The monthly PB positivity rate varied considerably, peaking in December (32.26%) and reaching its minimum in February (0.00%) (Figure 2).

**Table 1:** Demographic characteristics of children with SMPP in the non-PB and PB groups.

Variables	Total (*N* = 510)	non-PB (*N* = 450)	PB(*N* = 60)	*χ* ^2^	*P*-value
Gender, *n* (%)					
Male	250 (49.02)	226 (50.22)	24 (40.0)	2.214	0.137
Female	260 (50.98)	224 (49.78)	36 (60.0)		
Age distribution, *n* (%)					
age≤1 year	3 (0.59)	3 (0.67)	0 (0)	2.023	0.568
1 year < age≤3 years	16 (3.14)	15 (3.33)	1 (1.67)		
3 years < age≤6 years	112 (21.96)	101 (22.44)	11 (18.33)		
6 years < age≤16 years	379 (74.31)	331 (73.56)	48 (80.00)		
Season, *n* (%)					
Spring	73 (14.31)	65 (14.44)	8 (13.33)	10.998	0.012
Summer	209 (40.98)	195 (43.33)	14 (23.33)		
Autumn	171 (33.53)	144 (32.00)	27 (45.00)		
Winter	57 (11.18)	46 (10.22)	11 (18.33)		

### Radiological and bronchoscopic images of plastic bronchitis

**Figure 3:**
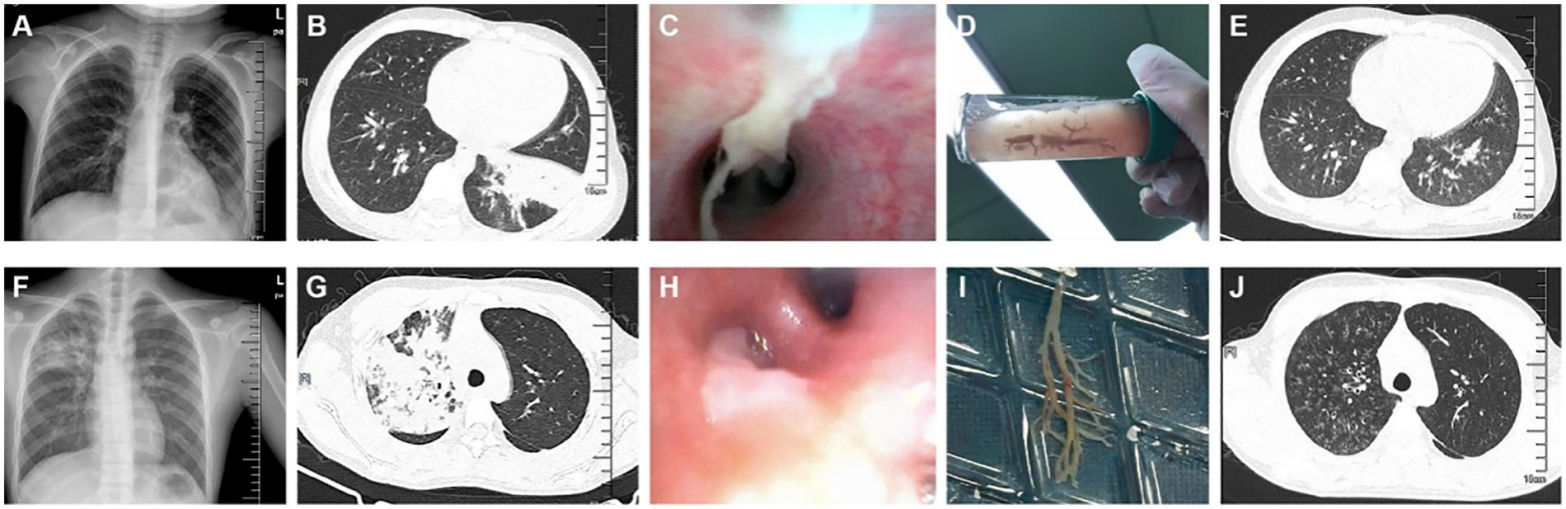
Radiological and bronchoscopic images of plastic bronchitis. Images A–E correspond to a 6-year-3-month-old boy diagnosed with SMPP. **(A)** Admission chest radiograph shows reticular and patchy opacities with blurred margins in the left lower lung; **(B)** Chest CT reveals extensive consolidative inflammation in the left lung; **(C)** Bronchoscopy demonstrates a mucus plug obstructing the left lower lobe bronchus; **(D)** The PB mucus plug is successfully extracted; **(E)** Follow-up chest CT at 2 weeks shows near-complete resolution of pulmonary inflammation and full re-expansion of the lung. Images F–J correspond to a 7-year-6-month-old boy diagnosed with SMMP. **(F)** Admission chest radiograph reveals a large area of increased opacity in the right upper lung; **(G)** Chest CT demonstrates a high-density shadow with atelectasis in the right upper lobe; **(H)** Bronchoscopy shows a mucus plug occluding the right upper lobe bronchus; **(I)** The mucus plug is successfully removed; **(J)** Follow-up CT at 10 days indicates complete pulmonary re-expansion and near-total resolution of inflammation in the right lung.

PB was managed by direct removal of bronchial casts under FB using suction and forceps, supplemented by adjunctive nebulized therapies such as mucolytics or bronchodilators as clinically warranted. Figure 3 depicts the characteristic radiological and bronchoscopic features of PB in two pediatric cases of SMPP, with consolidation affecting the left and right lungs, respectively.Case 1 was a 6-year-3-month-old boy. His imaging and bronchoscopic findings were as follows: the admission chest radiograph (A) showed reticular and patchy opacities with blurred margins in the left lower lung; chest CT (B) revealed extensive consolidative inflammation in the left lung; bronchoscopy (C) identified a mucus plug obstructing the left lower lobe bronchus; after removal of the PB mucus plug (D), follow-up CT at 2 weeks (E) demonstrated nearly complete resolution of pulmonary inflammation and full re-expansion of the lung. Case 2 was a 7-year-6-month-old boy. His imaging and bronchoscopic presentation included: the admission chest radiograph (F) showed a large area of increased opacity in the right upper lung; chest CT (G) demonstrated a high-density shadow with associated atelectasis in the right upper lobe; bronchoscopy (H) revealed a mucus plug occluding the right upper lobe bronchus; following successful removal of the mucus plug (I), follow-up CT at 10 days (J) indicated complete lung re-expansion and near-total resolution of inflammation in the right lung.

### Clinical manifestations

Among the analyzed clinical symptoms, the fever days were significantly longer in the PB group [8 (7∼10) days] than in the non-PB group [6 (4∼8) days; *P* < 0.001]. No significant differences were observed between the two groups in terms of cough duration, wheezing, dyspnea, or chest pain (*P* > 0.05). The incidence of runny nose was higher in the PB group compared to the non-PB group (*P* = 0.036). Regarding pulmonary signs, the PB group showed a significantly higher frequency of diminished breath sounds (*P* = 0.006), whereas the groups showed no significant differences in the presence of lung moisture rales, lung wheezing rales, or no rales (*P* > 0.05). For extrapulmonary manifestations, the PB group showed a higher prevalence of abnormal liver function and coagulation function (*P* < 0.001), while the incidence of pericardial effusion and rash did not differ significantly between groups. Furthermore, the PB group had a longer hospital stay (*P* < 0.001) and underwent more bronchoscopic interventions (*P* < 0.001) (Table 2).

**Table 2:** Clinical features of SMPP children in the non-PB and PB groups.

Variables	Total (*N* = 510)	non-PB (*N* = 450)	PB (*N* = 60)	χ^2^/Z	*P*-value
Clinical symptoms					
Fever days, M(P_25_∼P_75_)/d	7.00 (4.00∼8.00)	6.00 (4.00∼8.00)	8.00 (7.00∼10.00)	−5.239	<0.001
Cough days, M(P_25_∼P_75_)/d	7.00 (5.00∼10.00)	7.00 (5.00∼10.00)	7.50 (6.25∼10.00)	−0.885	0.376
Wheezing, *n* (%)	112 (21.96)	15 (3.33)	2 (3.33)	0.000	1.000
Runny nose, *n* (%)	21 (4.12%)	15 (3.33)	6 (10.00)	4.391	0.036
Dyspnea, *n* (%)	2 (0.39)	1 (0.22)	1 (1.67)	0.339	0.561
Chest pain, *n* (%)	16 (3.14)	11 (2.44)	4 (6.67)	1.992	0.158
Pulmonary signs, *n* (%)					
Lung moisture rales, *n* (%)	216 (42.35)	190 (42.22)	26 (43.33)	0.027	0.870
Lung wheezing rales, *n* (%)	28 (5.49)	23 (5.11)	3 (5.00)	0.529	0.467
No rales, *n* (%)	280 (54.90)	246 (53.67)	34 (56.67)	0.086	0.770
Diminished breath sounds, *n* (%)	137 (26.86)	112 (24.89)	25 (41.67)	7.585	0.006
Extrapulmonary manifestations					
Abnormal liver function, *n* (%)	28 (5.49)	15 (3.33)	13 (21.67)	30.851	<0.001
Pericardial effusion, *n* (%)	4 (0.78)	3 (0.67)	1 (1.67)	0.002	0.963
Abnormal coagulation function, *n* (%)	27 (5.29)	11 (2.44)	16 (26.67)	57.214	<0.001
Rash, *n* (%)	10 (1.96)	7 (1.56)	3 (5.00)	1.721	0.190
Past medical history					
Rhinitis, *n* (%)	220 (43.14)	199 (44.22)	21 (35.00)	1.836	0.175
Wheezing, *n* (%)	29 (5.68)	26 (5.78)	3 (5.00)	0.000	1.000
Allergies, *n* (%)	26 (5.10)	21 (4.67)	5 (8.33)	0.811	0.368
Length of hospital stay	8.00 (6.00∼9.00)	8.00 (6.00∼9.00)	11.00 (9.00∼14.00)	−8.598	<0.001
Number of bronchoscopic interventions	1.00 (1.00∼1.00)	1.00 (1.00∼1.00)	2.00 (2.00∼2.75)	−14.712	<0.001

### Laboratory tests and chest imaging

Significant intergroup differences were noted in laboratory findings. Hematological examination indicated that the PB group had a higher neutrophil percentage, alongside lower lymphocyte percentage and platelet count (all *P* < 0.001). Serum biochemical testing further demonstrated elevated levels of CRP, ALT, AST, LDH, and D-dimer in the PB group (all *P* < 0.001). No remarkable differences in WBC count,eosinophil count, eosinophil percentage and fibrinogen, were observed between the two groups. For chest imaging, the PB group showed higher rates of atelectasis, pleural effusion, and involvement of ≥2 lung lobes compared to the non-PB group, with the differences in atelectasis and pleural effusion reaching statistical significance (Table 3).

**Table 3:** Laboratory examination results and chest imaging of SMPP children in the non-PB and PB groups.

Variables	Total (*N* = 510)	non-PB (*N* = 450)	PB (*N* = 60)	χ^2^/Z	*P*-value
Laboratory findings					
WBC count, M(P25∼P75)/×109·L^−1^	8.33 (6.54∼10.61)	8.38 (6.57∼10.75)	8.12 (6.21∼10.25)	−1.445	0.149
Neutrophil percentage, M(P25∼P75)/%	64.80 (56.98∼72.53)	63.80 (56.3∼71.33)	72.75 (64.90∼79.88)	−5.175	<0.001
Lymphocyte percentage, M(P_25_∼P_75_)/%	25.70 (18.50∼32.50)	26.45 (19.78∼33.20)	17.80 (12.80∼24.13)	−5.878	<0.001
Eosinophil count, M(P_25_∼P_75_)/×10^9^·L^−1^	0.05 (0.01∼0.14)	0.05 (0.01∼0.14)	0.06 (0.02∼0.12)	−0.723	0.470
Eosinophil percentage, M(P_25_∼P_75_)/%	0.50 (0.10∼1.30)	0.50 (0.10∼1.30)	0.60 (0.20∼1.50)	−1.392	0.164
Platelet count, (P_25_∼P_75_)/×10^9^·L^−1^	300.00 (231.75∼375.25)	309.50 (242.00∼379.25)	249.50 (204.75∼318.75)	−3.959	<0.001
CRP, M(P_25_∼P_75_)/mg·L^−1^	8.20 (4.81∼20.31)	7.76 (4.24∼19.24)	22.07 (8.05∼36.52)	−4.868	<0.001
ALT, M(P_25_∼P_75_)/U·L^−1^	16.10 (11.90∼21.90)	15.50 (11.50∼20.00)	27.95 (17.90∼46.57)	−6.536	<0.001
AST, M(P_25_∼P_75_)/U·L^−1^	28.40 (22.70∼36.15)	27.50 (22.25∼34.20)	40.00 (29.10∼55.33)	−6.353	<0.001
LDH, M(P_25_∼P_75_)/U·L^−1^	312.60 (267.10∼396.55)	304.70 (264.75∼366.80)	516.25 (399.13∼628.80)	−8.365	<0.001
Fibrinogen, M(P_25_∼P_75_)/g·L^−1^	4.42 (3.86∼5.09)	4.38 (3.84∼5.08)	4.55 (3.88∼5.13)	−0.870	0.384
D-Dimer, M(P_25_∼P_75_)/ug·L^−1^	550.00 (360.00∼1100.00)	500.00 (340.00∼870.00)	1,905.00 (1,507.50∼4,402.50)	−9.246	<0.001
Chest imaging					
Atelectasis, n(%)	62 (12.16)	44 (9.78)	18 (30.00)	20.273	<0.001
Pleural effusion, n(%)	80 (15.69)	46 (10.22)	34 (56.67)	86.346	0.000
Emphysema	1 (0.20)	1 (0.22)	0 (0)	0.134	0.715
Involvement of ≥2 lung lobes, n（%）	250 (49.02)	217 (48.22)	33 (55.00)	0.324	0.973

Significant intergroup differences were noted in laboratory findings. Hematological examination indicated that the PB group had a significantly higher neutrophil percentage (72.75% vs. 63.80%), alongside lower lymphocyte percentage (17.80% vs. 26.45%) and platelet count (249.50 vs. 309.50 × 10⁹/L) (all *P* < 0.001). Serum biochemical testing further demonstrated markedly elevated levels of CRP (22.07 vs. 7.76 mg/L), ALT (27.95 vs. 15.50 U/L), AST (40.00 vs. 27.50 U/L), LDH (516.25 vs. 304.70 U/L), and D-dimer (1,905.00 vs. 500.00 μg/L) in the PB group (all *P* < 0.001). No remarkable differences in WBC count, eosinophil parameters, or fibrinogen were observed. For chest imaging, the PB group showed higher rates of atelectasis, pleural effusion, and involvement of ≥2 lung lobes compared to the non-PB group, with the differences in atelectasis and pleural effusion reaching statistical significance (Table 3).

### Multivariate regression analysis of PB in SMPP

Following univariate screening, significant variables were included in a multivariate analysis to identify independent risk factors for PB in SMPP. The model confirmed LDH ([OR] = 1.005, 95% confidence interval [CI]: 1.002∼1.007), pleural effusion (OR = 4.553; 95% CI: 2.261∼9.167), and length of hospital stay (OR = 1.278; 95% CI: 1.140∼1.433) as independent predictors. Notably, pleural effusion emerged as the strongest independent risk factor, associated with a 4.553-fold increased probability of PB development (Table 4).

**Table 4:** Multivariate logistic regression of risk factors for SMPP.

Variables	*β*	SE	Waldχ^2^	*P*-value	OR	95%CI
LDH (U/L)	0.005	0.001	16.790	0.001	1.005	1.002∼1.007
Pleural effusion	1.516	0.357	18.013	<0.001	4.553	2.261∼9.167
Length of hospital stay	0.245	0.058	17.649	0.001	1.278	1.140∼1.433
Constant	−6.673	0.680	96.182	<0.001	0.001	

### ROC curve

**Figure 4:**
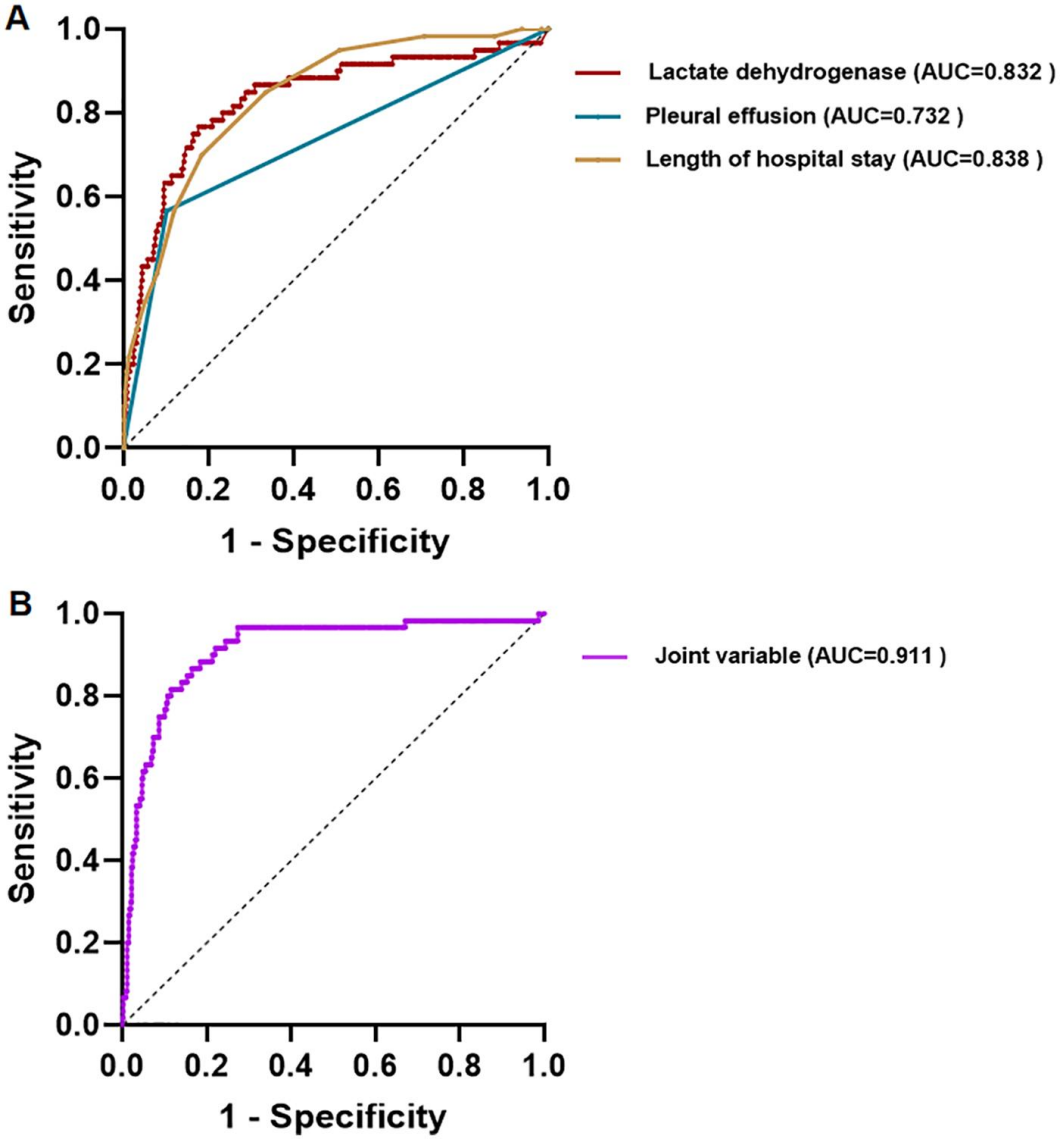
ROC curve for PB in SMPP. **A**: ROC curve of LDH, pleural effusion and length of hospital stay; **B**:ROC curve of joint variable.

Receiver operating characteristic (ROC) curve analysis identified LDH, pleural effusion, and length of hospital stay as significant predictors of PB in children with SMPP. Patients with LDH ≥ 397.60 U/L, hospital stay ≥ 9.50 days, or pleural effusion had a significantly increased risk of developing PB. Furthermore, the combination of these three indicators substantially improved predictive accuracy, yielding an area under the ROC curve (AUC) of 0.911 (95% CI: 0.868–0.953) (*P* < 0.001)(Table 5, Figure 4).

**Table 5:** Cut-off value of ROC curve for PB in SMPP.

Index	AUC (95%CI)	Cut-off	Specificity	Sensitivity	*P*-value
LDH (U/L)	0.832 (0.769∼0.896)	397.60	0.824	0.777	<0.001
Pleural effusion	0.732 (0.654∼0.811)	0.50	0.900	0.570	<0.001
Length of hospital stay	0.838 (0.788∼0.888)	9.50	0.816	0.700	<0.001
Joint variable	0.911 (0.868∼0.953)	0.11	0.835	0.867	<0.001

## Discussion

This study enrolled a total of 510 children with SMPP. Among them, 60 developed secondary PB, with an incidence rate of 11.76%. Epidemiological analysis revealed that the peak prevalence of SMPP in the local region occurred during summer and autumn, while the detection rate of secondary PB was highest in winter (19.30%), suggesting that seasonal variation may be an important factor influencing the development of PB secondary to SMPP.

MP represents a unique pathogen, taxonomically distinct from typical bacteria and viruses (18). This organism exhibits a global distribution across diverse climatic zones, demonstrating both sporadic cases year-round and periodic epidemic cycles, typically characterized by outbreaks every few years (19). Following the adjustment of COVID-19 containment measures, a significant resurgence of MP infections has been observed in multiple regions worldwide, accompanied by increased clinical severity and higher complication rates compared to previous epidemic cycles (20). Since 2023, large-scale outbreaks of MPP have been reported among children in various parts of the world, with a notable rise in the proportion of SMPP cases and complication rates (21). The drivers underlying this epidemiological pattern are not fully elucidated but are hypothesized to involve the interplay between the pathogen's intrinsic cyclic behavior and the “immunity gap” associated with the relaxation of non-pharmaceutical interventions (NPIs) (22, 23). The pathogenesis of SMPP may lie in the synergistic interplay between the MP pathogen itself and the excessive immune response triggered by its “advanced antigens,” which may collectively lead to life-threatening disease progression (24, 25). This study revealed that children aged 6–16 years represent a high-risk group for developing PB secondary to SMPP, accounting for 80% of cases. Although MPP is more common in school-aged children (26), Ding et al. have identified that SMPP is found mainly in the preschool population (7). This susceptibility may be associated with a propensity in this age group to mount an excessive immune-inflammatory response post-infection, leading to severe airway injury (27).

This study found that PB in SMPP children correlated with aggravated systemic inflammation, pulmonary complications, and multi-system involvement, manifested specifically as: prolonged fever duration and significantly elevated inflammatory markers (neutrophil percentage, CRP), suggesting a more intense inflammatory response; a higher incidence of atelectasis and pleural effusion on imaging, indicating worse airway obstruction and lung injury; and significantly increased levels of liver enzymes (ALT, AST) and D-dimer, demonstrating hepatic and coagulation system involvement. Current evidence indicates that children with SMPP exhibit a significant systemic inflammatory response. In pediatric patients with CAP, serum levels of inflammatory cytokines such as IL-6, IL-10, and IFN-*γ* are markedly elevated in MPP cases compared to non-severe pneumonia groups (28). When MP infection is complicated by PB, it can be accompanied by aberrant elevations in cytokines such as IL-1β, IL-8, IL-2, and IL-10 (13). Children with SMPP exhibit significantly elevated levels of systemic inflammation and tissue injury markers, including the granulocyte percentage, CRP, D-dimer, and LDH, while their BALF is predominantly characterized by neutrophil infiltration (29). CRP, an acute-phase protein synthesized by the liver in response to infection, rises rapidly within hours and serves as a reliable indicator for assessing the severity and complication risk of pediatric pneumonia (30). D-dimer, a fibrin degradation product and a widely used clinical marker for coagulation activation (31), has been shown by Jin et al. to have plasma levels that are positively correlated with the severity of severe pneumonia (32). Zhang et al. proposed that a D-dimer level >0.64 mg/L combined with an LDH level >379 U/L holds significant value for the assessment of MPP (33).

Our study confirmed that although most cases of SMPP-associated PB were mild and responsive to a single FB procedure, the overall increased intervention requirement in the PB group was attributable to a minority of refractory cases, revealing the condition's substantial complexity and persistent nature. PB presents with diverse etiologies, including infectious (e.g., Mycoplasma pneumoniae) and non-infectious causes (34–36). This etiological heterogeneity necessitates a broad spectrum of clinical management strategies, including inhaled medications, systemic therapies, and bronchoscopic interventions. The significant variation in both etiology and required management underscores that treatment outcomes are closely linked to the underlying cause (37). However, because FB enables the simultaneous identification of PB and clearance of airway secretions, it remains a crucial intervention for the early improvement of alveolar ventilation in patients with SMPP complicated by PB (15). This study multivariable logistic regression analysis identified elevated LDH levels, pleural effusion, and length of hospital stay as independent risk factors for PB in children. The combination of these three indicators demonstrated significantly enhanced predictive value. LDH, a widely distributed enzyme in myocardial, hepatic, skeletal muscle, and pulmonary tissues, is associated with a significantly increased mortality risk in patients with pulmonary infections when its level exceeds 900 U/L (38). Furthermore,in our case series of SMPP, the incidence of pleural effusion was 56.67% in the PB group, significantly higher than the 10.22% observed in the non-PB group. This is consistent with the findings of Zhang et al., who also reported a significantly higher incidence of pleural effusion in PB cases associated with refractory MPP (39). Both studies identify pleural effusion and elevated LDH as critical risk factors. While the model developed by Zhang et al. focuses on predicting PB based on pre-bronchoscopic clinical features, our study further identifies prolonged hospitalization as an independent risk factor, which may help in recognizing more severe or protracted cases. Together, these results support the use of combined clinical indicators for the early recognition of high-risk SMPP patients, thereby facilitating timely bronchoscopic intervention.

## Conclusions

This study identifies elevated serum LDH, pleural effusion, and length of hospital stay as a clinically actionable composite marker for predicting PB in children with SMPP. By applying these readily available parameters, clinicians can more effectively identify high-risk patients who may benefit from intensified monitoring and timely bronchoscopic intervention. While offering a practical framework for in-hospital risk stratification, this study acknowledges the limitations inherent to its single-center retrospective design, which include potential cohort imbalance and the inability to establish causality or evaluate long-term outcomes. Consequently, future research should prioritize multicenter prospective validation to confirm the model's generalizability and integrate mechanistic investigations to elucidate the pathophysiological pathways linking these clinical indicators to PB development, thereby advancing targeted preventive and therapeutic strategies.

## Data Availability

The original contributions presented in the study are included in the article, further inquiries can be directed to the corresponding authors.
